# Coordination dynamics of thoracic and abdominal movements during voluntary breathing

**DOI:** 10.1038/s41598-022-17473-9

**Published:** 2022-08-02

**Authors:** Mimu Higashino, Kohei Miyata, Kazutoshi Kudo

**Affiliations:** 1 Graduate School of Interdisciplinary Information Studies, The University of Tokyo, 3-8-1, Komaba, Meguro, Tokyo, 153-8902 Japan; 2 Graduate School of Arts and Sciences, The University of Tokyo, 3-8-1, Komaba, Meguro, Tokyo, 153-8902 Japan

**Keywords:** Human behaviour, Respiration

## Abstract

Thoracic and abdominal movements can be tightly coupled during voluntary breathing, such as when singing and playing wind instruments. The present study investigated the coordination of thoracic and abdominal movements during voluntary breathing using a dynamical systems approach. We examined whether there are two stable coordination patterns, and if the coordination pattern would abruptly change when the breathing frequency increased, which is known as phase transition. The participants inhaled and exhaled repeatedly at 7.5, 15, 30, 60, or 120 breaths per minute. At the beginning and end of the experiment, the participants performed breathing at their preferred frequency. As a result, the coordination pattern at the lower and preferred frequencies exhibited an in-phase pattern. When breathing frequency increased, participants showed deviated coordination patterns from the in-phase pattern to either a thoracic-leading pattern, an abdominal-leading pattern, or an anti-phase pattern depending on the individual. These deviations occurred gradually; thus, phase transition was not observed. Our findings suggest that thoracic and abdominal movements are tightly coupled at lower frequencies, but their patterns vary depending on the breathing frequency and individuals. Therefore, the present study suggests the importance of viewing breath control in terms of coordination of thoracic and abdominal movements.

## Introduction

Breathing—inhalation and exhalation—is the basis of all human activity. Although we usually breathe involuntarily, we also do it voluntarily when we sing, play wind instruments, or exercise^1^. It is well known that being aware of thoracic and abdominal movements is important for breath control. These two movements are coordinated rather than controlled independently because the movement of the diaphragm, a dome-shaped skeletal muscle that forms the bottom of the chest cavity, affects both the thoracic and abdominal cavities.

The diaphragm and external intercostal muscles are the major inhalation muscles. When the diaphragm contracts, it flattens, and the dome descends, thus increasing the vertical length of the chest cavity. When the external intercostal muscles contract, the ribs are elevated. As a result, the diameter of the thoracic cavity increases in the anterior–posterior and lateral directions. Breathing that relies mainly on movement of the diaphragm is deep, and is called a diaphragmatic breathing pattern. On the other hand, breathing mainly through the excursion of the rib cage is shallow, and is called a costal breathing pattern. Normal, quiet breathing involves both breathing patterns. During quiet breathing, the contraction of the diaphragm and external intercostal muscles causes the thoracic cavity to expand, which reduces intra-thoracic pressure and allows air to flow into the lungs. The diameter of the abdominal cavity also increases in the horizontal, anterior, and posterior directions, because the intra-abdominal pressure increases as the diaphragm contracts. Therefore, both thoracic and abdominal diameters increase in the anterior and posterior directions during inhalation and decrease during exhalation. It has been reported that the lag between thoracic and abdominal movements (thoraco-abdominal asynchrony) is observed in many respiratory disorders and/or respiratory muscle disfunctions^2–4^. Therefore, it is important to view breath control in terms of thoracic and abdominal movement coordination.

One of the major approaches to estimate human movement coordination is the dynamical systems approach. The dynamical systems approach has been used to explain the “self-organized” pattern formation in human movement coordination. This approach has successfully modeled the spatial and temporal patterns in nonequilibrium physical and chemical systems that emerge spontaneously^5–7^. Kelso first employed this approach to estimate human movement coordination^7^. In his experiment, participants oscillated their index fingers bilaterally in the transverse plane, that is, abduction–adduction. He found different coordination patterns depending on the movement frequency. When the movement frequency was low, participants could move their index fingers both in an in-phase (moving toward and away from each other) pattern and anti-phase (moving parallel) pattern. However, when the movement frequency was increased, participants abruptly could not move their index finger in the anti-phase pattern, even though they could in the in-phase pattern. Thus, an abrupt transition occurs from the anti-phase pattern to the in-phase pattern at a critical frequency, but not vice versa. This transition is called phase transition and is often taken as the landmark feature of a dynamical system. In addition to movement frequency, it has been demonstrated that the difference between the preferred frequencies of the two movements (i.e., detuning) caused the shift away from an in-phase and anti-phase pattern and decreased the stability of coordination^8–11^. These phenomena were successfully modeled by defining the relative phase between two movements as an order parameter, and frequency as a control parameter^12^. This model is known as the Haken-Kelso-Bunz (HKB) model.

The coordination dynamics have been shown in contexts ranging from inter-limb^10,12,13^, intra-limb^14–16^, postural^17^, sensorimotor coordination^18^ to interpersonal coordination^8,9,19–21^. This ubiquity of the coordination dynamics in various human movements led us to hypothesize that the thoracic and abdominal coordination also has two attractors—the in-phase and anti-phase patterns—and the breathing frequency is the control parameter. That is, the phase relation between thoracic and abdominal movements is close to 0° or 180°. When the relative phase is close to 0°, the chest expands along with the abdomen. On the other hand, when the relative phase is close to 180°, the chest and abdomen expand alternately. This hypothesis is supported by the fact that when its frequency increases, breathing becomes shallow, thus primarily relying on the costal breathing pattern. During voluntary costal breathing, the abdominal compartment is extended in the cranial-caudal direction when the rig cage is elevated for inspiration, which results in an inward abdominal motion. The shortened abdominal compartment when the rib cage goes down for expiration results in outward abdominal motion. By primarily relying on costal breathing, the breathing pattern would be the anti-phase pattern. The phase transition between the two patterns is thus expected to occur when the breathing frequency changes. Although previous studies have demonstrated the coordination of breathing cycles with steps during locomotion^22,23^ and vocalization with whole-body movement^24^, the voluntary breathing coordination between thoracic and abdominal movements depending on breathing frequency remains unknown. The dynamical system approach is expected to provide a framework to model thoraco-abdominal coordination during breathing, which enables us to verify the possibility that a general principle of coordination is manifested in thoraco-abdominal coordination and propose a method for quantifying coordination pattern characteristics.

The purpose of this study was to examine whether thoracic and abdominal coordination during voluntary breathing obeys the coordination dynamics. We investigated the coordination pattern of thoracic-abdominal movements depending on the breathing frequency. We examined whether the phase relation between thoracic and abdominal movements has two stable patterns and abrupt changes between the two patterns when breathing frequency changes. Furthermore, we expected that the frequencies at which phase transition occurs varied depending on individual’s preferred frequency of breathing and subjective feelings of speed and difficulty. Thus, we also recorded thoraco-abdominal coordination at the preferred frequencies and asked participants to rate the subjective speed and difficulty of each experimental frequency.

## Results

### Breathing at preferred frequency

The mean and standard deviation (SD) of preferred breathing frequencies was 12.38 ± 3.71 breaths per minute (bpm) (Mean ± SD) at the beginning of the experiment, and 10.65 ± 3.16 bpm after the experiment. The breathing frequency was significantly lower at the end of the experiment, *t*(14) = 2.36, *p* = 0.034. The mean breathing frequency across the beginning and end of the experiments was 11.51 ± 3.50 bpm. The mean and SD of relative phase angles between thoracic and abdominal movements at preferred frequency was − 6.42 ± 12.48° at the beginning of the experiment and − 4.73 ± 13.17° after the experiment, indicating that participants breathed in the in-phase pattern. There was no significant difference between the beginning and the end of the experiment, *t*(14) = 0.59, *p* = 0.567. The mean relative phase angle across the beginning and end of the experiment was − 5.58 ± 12.63°.

### Subjective speed and difficulty scores

**Figure 1:**
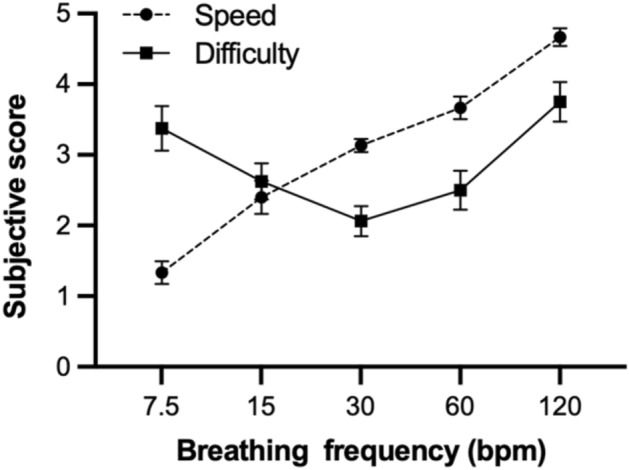
Subjective speed and difficulty scores. Vertical bars represent between-subject standard error.

Figure 1 shows the results of the subjective speed and difficulty scores as a function of breathing frequency. One-way analysis of variance (ANOVA) with a within-subject factor of breathing frequency on the subjective speed score showed a significant main effect, *F*(1.78, 24.94) = 87.70, *p* < 0.001, ${\eta }_{G}^{2}$ = 0.778. Post-hoc tests revealed that the subjective speed score significantly increased as breathing frequency increased, *ps* < 0.007. The ANOVA on the subjective difficulty score also showed a significant main effect of breathing frequency, *F*(2.26, 33.95) = 7.47, *p* = 0.001, ${\eta }_{G}^{2}$ = 0.256. Post-hoc tests revealed that the subjective difficulty score for 120 bpm was higher than that for 15, 30, and 60 bpm, *ps* < 0.017. The difficulty score for 7.5 bpm was higher than that for 30 bpm, *p* = 0.004.

### Phase relation between thoracic and abdominal movements

**Figure 2:**
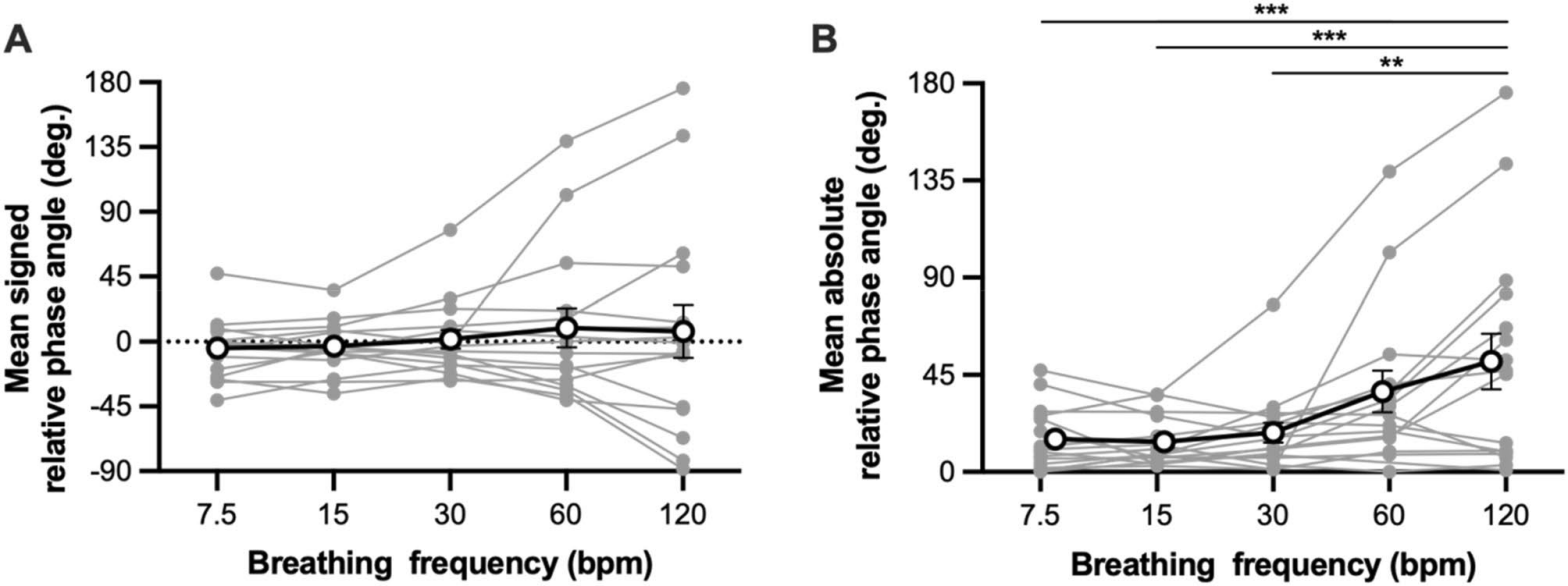
Mean signed and absolute relative phase angle between thoracic and abdominal movements. Solid markers represent individual participants. Open circles indicate mean value across participants. Vertical bars represent between-subject standard error. **p < 0.01, ***p < 0.001.

**Figure 3:**
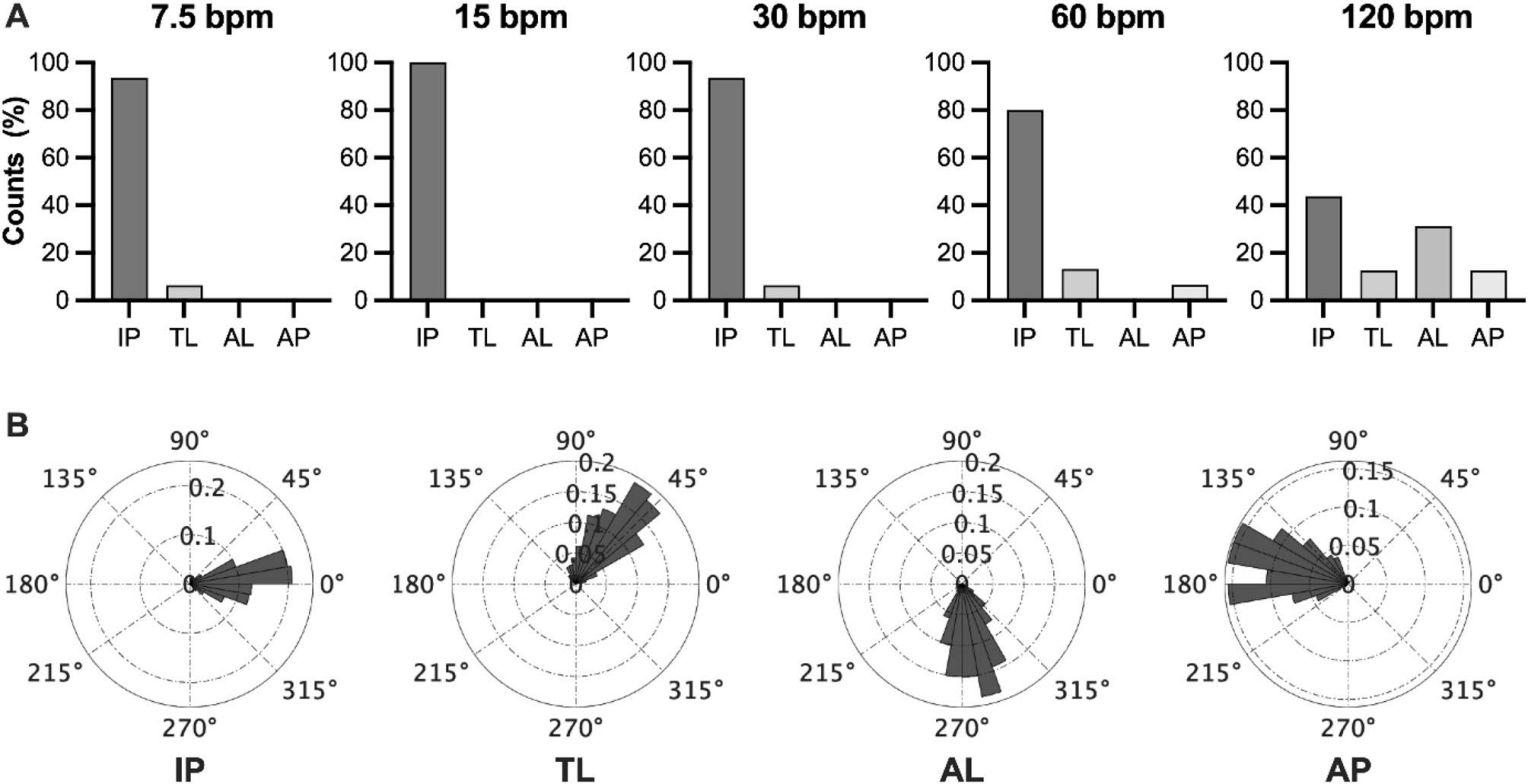
Frequency of particular thoraco-abdominal coordination patterns identified by the phase relation analysis, and a representative example of a participant’s relative phase histogram for each pattern. *IP* in-phase pattern, *TL* thoracic-leading pattern, *AL* abdominal-leading pattern, *AP* anti-phase pattern.

Figure 2-A shows the mean relative phase angle between thoracic and abdominal movements as a function of breathing frequency. The ANOVA showed no significant main effect of breathing frequency, *F*(1.16, 16.21) = 0.68, *p* = 0.442. This is because some participants showed the thoracic-leading pattern, while others showed abdominal-leading pattern. The histograms of relative phase angles for each participant are shown in Supplementary Fig. S1. We divided the phase relation into four distinct patterns (in-phase, thoracic-leading, abdominal-leading, and anti-phase patterns). At 120 bpm, seven participants (43.8%) showed the in-phase pattern, two participants (12.5%) showed the thoracic-leading pattern, five participants (31.3%) showed the abdominal-leading pattern, and two participants (12.5%) showed the anti-phase pattern (Fig. 3).

To examine whether the phase angle deviated from the in-phase pattern when the breathing frequency increased, the relative phase angle was converted into absolute values (Fig. 2B). The ANOVA on the absolute value of relative phase angle showed a significant main effect of breathing frequency, *F*(1.28, 17.91) = 7.77, *p* = 0.008, ${\eta }_{G}^{2}$ = 0.211. Post-hoc tests revealed that the relative phase angle at 120 bpm was higher than those at 7.5, 15, and 30 bpm, *p* < 0.001, *p* < 0.001, and *p* = 0.001, respectively. Thus, the relative phase angle deviated from the in-phase pattern when the breathing frequency increased.

**Figure 4:**
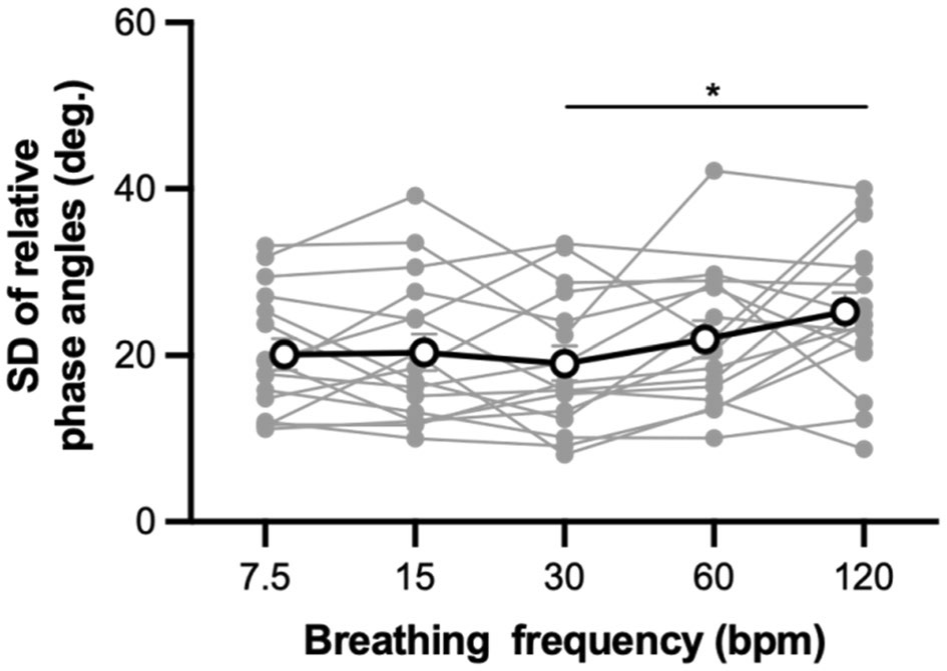
SD of relative phase angles between thoracic and abdominal movements. Solid markers represent individual participants. Open circles indicate mean value across participants. Vertical bars represent between-subject standard error. *p < 0.05.

Figure 4 shows the SD of the relative phase angle as a function of breathing frequency. The ANOVA on the SD of the relative phase angles showed a significant main effect of breathing frequency, *F*(3.19, 44.73) = 3.27, *p* = 0.027, ${\eta }_{G}^{2}$ = 0.082. Post-hoc tests revealed that the SD of the relative phase angle for 30 bpm was lower than those for 120 bpm, *p* = 0.019.

## Discussion

The aim of the present study was to test the hypothesis that coordination between thoracic and abdominal movements during voluntary breathing obeys the dynamics governing various human movement coordination. In our study, the participants performed voluntary breathing at various frequencies. We hypothesized that there are two stable coordination patterns (in-phase; thoracic cavity expands together with abdomen and anti-phase; thoracic cavity and abdomen expand alternately) and that the phase pattern abruptly switches when breathing frequency changes. Our results showed that the phase relation was an in-phase pattern at lower breathing frequency and gradually changed to either the thoracic-leading, abdominal-leading, or anti-phase pattern when breathing frequency increased. These results suggest that the coordination dynamics of thoracic and abdominal movements differ from those observed in other types of human movement coordination, such as bimanual coordination, sensorimotor coordination, and interpersonal coordination.

The thoracic and abdominal coordination during breathing showed an in-phase pattern around the preferred frequency (11.51 ± 3.50 bpm), such as 7.5 and 15 bpm. This result is consistent with a previous study that found the relative phase between thoracic and abdominal movements was stably close to 0° in healthy infants^25^. As the diaphragm contracts and descends during the inhalation phase, the abdominal organs are pushed down so that the diameter of the abdominal cavity increases in the anterior–posterior and lateral directions along with the expansion of the chest cavity. In the exhalation phase, the stretched lungs and chest wall return to their resting shape due to elastic forces. The diameter of the abdominal cavity also shrinks because the viscera in the abdomen move upward as the diaphragm relaxes. Thus, it is natural that the abdominal and thoracic movements synchronize during the in-phase pattern.

Previous studies assessing the coordination dynamics of various human movements demonstrated that movement frequency acts as the control parameter responsible for changing the stability of system states; thus, certain coordination patterns are organized by manipulating the movement frequency^6,12^. Our results showed that the phase angle changed as a function of breathing frequency, suggesting that breathing frequency is the control parameter for thoracic and abdominal coordination. Therefore, breathing frequency plays a critical role in the organization of thoracic-abdominal coordination pattern.

When breathing frequency increased, the coordination pattern was not only in-phase but also asynchronous, such as the thoracic-leading pattern, the abdominal-leading pattern, and the anti-phase pattern. The groups also exhibited distinct quantitative characteristics (Fig. 3 and Supplementary Fig. S1). The IP group (n = 7) appears to have the least variability among the groups (Fig. 3B), suggesting it is the most stable pattern. The AP group (n = 2) seems to have more variability from the low breathing frequency (Supplementary Fig. S1). Therefore, the phase relation may change to an anti-phase pattern when the in-phase pattern is unstable, even at low frequency. The difference between the TL (n = 2) and the AL groups (n = 5) was simply whether the thoracic or the abdominal movement was ahead of the other, but the degree of phase shift was greater in the AL group (Fig. 3B). As the frequency was also higher in the AL group (Fig. 3A), the phase relation may be prone to an AL pattern when phase shift occurs. Although the number of participants in each group was small, our results showed clear individual differences.

The phase relation changed gradually and differently depending on individuals. These large individual differences are contrary to those of previous studies assessing various forms of human movement coordination^6,12^. It has been demonstrated that there are certain phase relations between movements which are especially stable (e.g., 0° and 180°), whereas other phase relations tend to be more variable^6,12^. When the movement frequency increases, the phase relation abruptly changes from one to the other coordination pattern. Compared to other human movement coordination, thoracic and abdominal coordination can be strongly influenced by internal demands, such as gas exchange. This characteristic might make thoracic-abdominal coordination different from other movement coordination. Additionally, we explored a limited range of breathing frequency; thus, our results may show the middle of the phase shift from the in-phase pattern to the anti-phase pattern. When exploring higher breathing frequencies, one might find an anti-phase pattern for all participants.

Another possible interpretation of the large individual differences in this study is the effect of tidal volume, or volume of each breath. In general, as the frequency of breathing increased, tidal volume decreased. This was also observed in our dataset (see Supplementary Fig. S2). Therefore, the rate of decrease in tidal volume at high frequencies differs between individuals with relatively small or large tidal volumes at low frequencies. Thus, it is possible that participants who had low tidal volume during low frequencies had a lower rate of decrease in tidal volume at high frequencies, and hence were less likely to change their thoracic-abdominal coordination. Elucidation of the cause of the individual differences, including the measurement of respiratory volume, is a subject for future research.

Fatigue during the experiment can disturb breathing coordination patterns. Breathing frequency can increase when the participants become tired. However, participants breathed slower at the end of the experiment than at the beginning of the experiment. Furthermore, the mean relative phase angles at the beginning and end of the experiment were both in-phase patterns. These results suggest that the participants performed the task stably during the experiment.

The subjective speed score linearly increased from 7.5 to 120 bpm, whereas the subjective difficulty score followed a U-shape pattern, with the lowest score at 30 bpm. The further the breathing frequency was from 30 bpm, the more difficult the subject seemed to find it. This tendency was also reported in a previous study of auditory-motor synchronization^18^. Miura et al.^18^ demonstrated a U-shape pattern with the lowest score at 100 bpm in subjective difficulty, indicating that participants felt that auditory-motor synchronization was more difficult at extremely low or high frequencies. These findings suggest that there is a comfortable frequency of movements, and that it becomes difficult when the frequency deviates from the comfortable frequency-either too slow or too fast. The results of our study indicate that 30 bpm is the most comfortable frequency for voluntary breathing.

In practical settings, such as singing and vocalizing for acting, there are two types of breathing methods: “thoracic breathing,” which mainly focuses on thoracic movement, and “abdominal breathing,” which consciously uses the abdominal movement. However, this classification may lead to misinterpretations that one focuses only on thoracic or abdominal movements. The thoracic and abdominal cavities are separated only by the diaphragm, meaning thoracic and abdominal movements affect each other. Therefore, these two movements should be coordinated, rather than independently controlled. The present study successfully identified this coordination pattern between thoracic and abdominal movements during voluntary breathing. Furthermore, this study demonstrated that the coordination pattern changes when breathing frequency increases, revealing individual variation in the change. These results suggest that it is important to consider the effects that breathing frequency and individual variations of coordination pattern have on the relationship between thoracic and abdominal movements in various performances.

Our study has some limitations. Our participants breathed via a harmonica so that they might inhale more strongly than usual. This could have affected the results of our study. In our study, the preferred frequency of breathing was 11.51 ± 3.50 bpm. Previous studies have suggested a wide range of spontaneous breathing frequencies (between 6 and 31 bpm) in human adults^26,27^. Thus, our experimental setting was not far from spontaneous breathing, even though the participants breathed via a harmonica. Furthermore, the purpose of breathing is to inhale and exhale air; hence, the amount of inhaled or exhaled air can be a factor affecting thoracic and abdominal coordination. However, in our experimental setting, we could not measure this amount. This warrants a future study evaluating the effect that the amount of inhaled and exhaled air has on the coordination pattern. Lastly, we explored a limited range of breathing frequencies in our experimental paradigm. There may be more anti-phase patterns when we observe coordination at frequencies higher than 120 bpm. However, fast breathing induces a lack of oxygen because the gas stays in the dead space in the lungs, hindering suitable gas exchange. Thus, future studies must take care to set the experimental breathing frequency within a reasonable range.

The main finding of this study is that the coordination of thoracic-abdominal movement during breathing has different coordination dynamics from inter-limb coordination. That is, as the movement frequency increased, the relative phase angle between the thoracic and abdominal movements gradually changed from the in-phase pattern to asynchronous coordination. This study demonstrated coordination patterns between thoracic and abdominal movements, depending on the breathing frequency. Breathing training that takes thoracic-abdominal coordination into account is important for educational support in singing, playing musical instruments, and many other performances.

## Methods

### Participants

Sixteen healthy volunteers (seven female and nine male), aged 22–35 years participated in this experiment. All participants were recruited from the University of Tokyo and had no self-reported breathing or hearing impairments. All participants signed an informed consent form prior to participation. This study was approved by the Ethics Committee Regarding Experiments and Surveys on Human Subjects at the University of Tokyo and was conducted in accordance with the Declaration of Helsinki.

### Experimental task and design

Since it is difficult to intentionally coordinate thoracic and abdominal movements without any training, we used a similar protocol to that of Bardy et al.^17^. Their study investigated the postural coordination of hip and ankle joints during body sway in the standing posture. The participants were not explicitly instructed to coordinate these joints, but to track a moving target with their head. By manipulating the frequency of the target movement, they demonstrated the phase transition of postural coordination modes. In our study, we asked participants to synchronize their breathing with metronome beats, then manipulated the frequency of the metronome.

**Figure 5:**
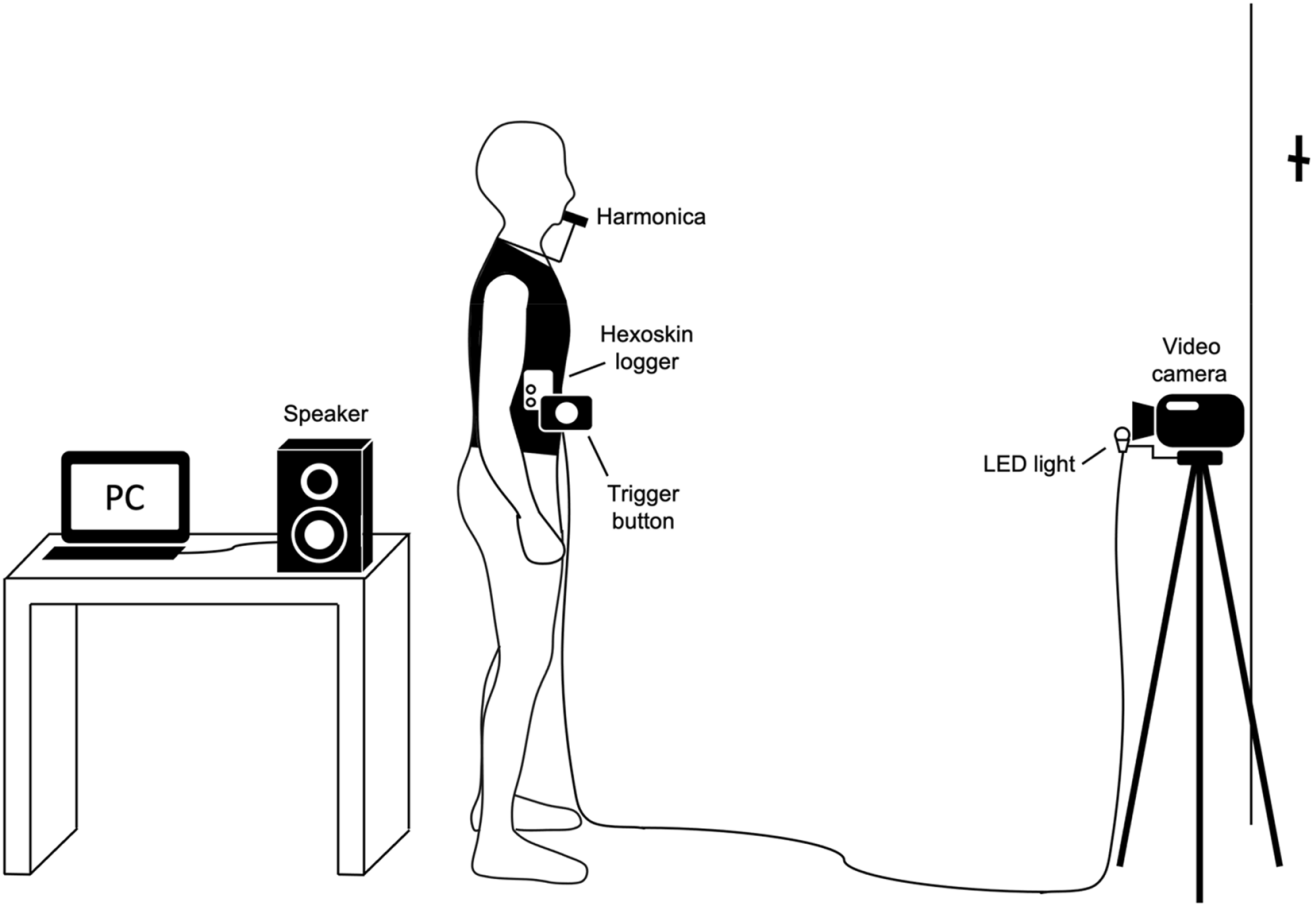
Experimental setup. Participants breathed via a harmonica while looking at a fixed point on the wall from a distance of 2 m. The thoracic and abdominal movements during breathing were measured using a Hexoskin vest equipped with two strain gauge bands around the chest and waist and an electrocardiogram sensor. The data was stored in a logger equipped with a 3-axis accelerometer. The metronome beats were presented via a speaker from a laptop. The sounds of the harmonica and metronome beats were recorded using a video camera. The Hexoskin data and video recordings were synchronized by hitting a trigger button that illuminated a light-emitting diode in the view of the video camera. The participants pressed the button on a Hexoskin logger with the accelerometer at the beginning of each trial.

The experimental task was voluntary breathing, that is, repeatedly inhaling and exhaling to metronome beats. To detect the onset and duration of breathing, participants were asked to breathe via a harmonica, which creates different sounds for inhalation and exhalation. During the task, participants were instructed to rest their arms at their sides, stand upright with their feet roughly at shoulder width, and look at a fixed point on the wall from a distance of 2 m (Fig. 5). Participants were asked to perform 12 cycles of breathing for each trial without holding their breath.

The breathing frequency ranged from 7.5 to 120 bpm. The frequency was manipulated in separate trials. Auditory metronome beats were presented to regulate breathing frequency. Each trial comprised 24 beats (two beats per breathing cycle) presented at rates of 15, 30, 60, 120, or 240 beats per minute. Participants were instructed to switch from inhalation to exhalation or from exhalation to inhalation when a metronome beat was presented. At the beginning of each trial, four additional beats served as ready cues (for two cycles of breathing). The duration of each condition was approximately 9 s at 120 bpm, 16 s at 60 bpm, 30 s at 30 bpm, 58 s at 15 bpm, and 114 s at 7.5 bpm.

Participants completed a total of 15 trials (five breathing frequencies × three repetitions). The trial order was randomized to avoid order effects. At the beginning and end of the experiment, participants also performed breathing at their preferred frequency for one minute without any pacing signals.

### Procedure

All experiments were conducted in a quiet experimental room at the Komaba Campus at the University of Tokyo. Participants first received an explanation about the experimental purpose and task. After participants gave their written informed consent to participate in the study, they were fitted with a properly sized wearable recorder (see “Apparatus and data collection”). The participants practiced the task for a few minutes before the experimental recording. They were instructed to synchronize breathing with the metronome beat as stably as possible. After the experiment, participants answered a questionnaire about their exercise experience and whether they smoked. They also rated the subjective speed and difficulty of each condition on a 5-point scale. It was assumed that individuals have different comfortable breathing frequencies and thus different impressions of the frequencies of the experimental conditions. Since this impression may be related to the frequency of the phase transition, we recorded these two subjective assessments. The entire experiment lasted approximately an hour.

### Apparatus and data collection

Figure 5 shows the experimental setup. The thoracic and abdominal movements during breathing were measured using a Hexoskin vest (Carré Technologies Inc., Canada) equipped with two strain gauge bands around the chest and waist and an electrocardiogram sensor. A small value of the strain gauges indicates shrinkage, and a large value indicates expansion. The vests were available in different sizes and styles in order to fit the participants’ bodies. The data was stored in a logger equipped with a 3-axis accelerometer.

To record the onset and duration of breathing, we asked the participants to inhale and exhale via a harmonica (AH-1020 G 10-hole harmonica, ARIA, Japan), which creates different sounds between inhaling and exhaling. The harmonica was fixed in front of the participants’ mouths with a harmonica holder.

The metronome beats were made using MATLAB R2020a (MathWorks, USA) and presented via a speaker (Foster Electric Company Ltd., Japan) from a MacBook Air (Apple, USA). The sounds of the harmonica and metronome beats were recorded using a video camera (HC-WX995M Digital 4 K video camera, Panasonic, Japan).

The sampling frequencies were 128 Hz for the strain gauges, 64 Hz for the accelerometer, 29.97 Hz for video, and 48 kHz for video audio. The Hexoskin data and video recordings were synchronized by hitting a trigger button that illuminated a light-emitting diode in the view of the video camera. The participants pressed the button on a Hexoskin logger with the accelerometer at the beginning of each trial. The luminance signals and the peak of acceleration were detected as cues to start the trial in the offline analysis. The data from each trial was then segmented using these cues.

### Data analysis

To quantify the relationship between the thoracic and abdominal movements, the relative phase angle between them was calculated. First, the thoracic and abdominal movements were smoothed and detrended using a band-pass filter to extract movement oscillation at the given frequencies. The pass frequencies were set from 0.1 to 0.3 Hz in 7.5 bpm, from 0.15 to 0.75 Hz in 15 bpm, from 0.25 to 1.5 Hz in 30 bpm, from 0.5 to 2.5 Hz in 60 bpm, and from 1.5 to 4.5 Hz in 120 bpm. The first two and the last cycles of breathing were discarded to remove transient effects and distortions at the beginning and end of filtered time-series because of the band-pass filter. To confirm movements oscillating at a given frequency, we performed Fast Fourier Transform (FFT) using the “FFT” function in MATLAB. If the frequency power of either thoracic or abdominal movement at a given frequency was less than one, the data was considered to be not oscillating and discarded. Velocities were then obtained by differential calculus of filtered displacement data of thoracic and abdominal movements.

Before calculating the relative phase angle, we performed half-cycle normalization to avoid phase angle distortion due to variations in oscillation center and movement frequency^28,29^. First, we defined half-cycles by detecting maximum and minimum peaks of girth of chest and waist respectively with the “findpeaks” function in MATLAB. The displacement data was normalized by centering per half-cycle by subtracting the center value between minimum and maximum peaks of the respective half-cycle bin^28^. The velocity was normalized by dividing each half-cycle of the velocity by $\pi /hp$, where $hp$ is the corresponding half period^29^. The normalized displacements and velocities were converted to a Z-value and plotted on a phase plane with the displacement data on the X-axis and the velocity data on the Y-axis, to compute the continuous phase of each movement.

The mean and SD of the relative phase angles between thoracic and abdominal movements were calculated using circular statistics^30^. The relative phase angle was calculated as the abdominal phase angle minus the thoracic phase angle; a positive value means thoracic-lead, while negative value means abdominal-lead. First, we calculated the mean and SD of the relative phase within a trial, then calculated the mean values across trials. The mean values across the trials were used as representative values for each participant.

Since our results indicated that the phase relation showed various patterns as the breathing frequency increased, we divided the phase relation into four distinct patterns (in-phase pattern, thoracic-leading pattern, abdominal-leading pattern, and anti-phase pattern). At 7.5 bpm (i.e., the lowest breathing frequency), the phase relation was the in-phase (mean relative phase angle was − 5.24°). The 2 SD of phase angles in the 7.5 bpm was 41.06°, so ± 45° from 0° was defined as the in-phase pattern. Similarly, the thoracic-leading pattern, abdominal-leading pattern, and anti-phase pattern were defined in the range of ± 45° from 90°, − 90°, and 180°, respectively.

The breathing data at the preferred frequency were analyzed in the same manner. The band-pass frequency was set from 0.1 to 4.5 Hz. The first 10 s and the last 5 s of data were discarded to remove transient effects and distortions at the beginning and end of filtered time-series. Velocities were then obtained by differential calculus of filtered displacement data of thoracic and abdominal movements. The calculation of the relative phase angle between thoracic and abdominal movements was same with that for trials where the breathing frequency was prescribed. The frequency of breathing was calculated as the interval between the maximum values of thoracic movements.

One trial and subjective speed score of a participant were not recorded because of a technical problem. Data of nine trials from two participants were discarded because of recording failure. Data of nine trials from five participants were discarded because oscillation was not confirmed in either thoracic or abdominal movement with the FFT analysis.

### Statistical analysis

A one-way ANOVA with a within-subject factor of breathing frequency (7.5, 15, 30, 60, and 120 bpm) was performed on (1) the subjective speed score, (2) the subjective difficulty score, (3) relative phase angle between thoracic and abdominal movements, and (4) SD of the relative phases. Paired t-tests were performed on (5) preferred breathing frequency and (6) relative phase angle between thoracic and abdominal movements at the preferred frequency. The Greenhouse–Geisser correction was used in cases where Mauchly’s test of sphericity was significant. The Holm correction was used for multiple comparisons in post-hoc tests. The statistical significance level was set at *p* < 0.05.

## Supplementary Information

Supplementary Figures.

## Data Availability

The datasets generated during and/or analyzed during the current study are available from the corresponding author on reasonable request.
