# Supplementary material for: Coordination dynamics of thoracic and abdominal movements during voluntary breathing
Source: Sci Rep. 2022 Aug 2;12:13266. doi: 10.1038/s41598-022-17473-9 (PMC9345990; doi:10.1038/s41598-022-17473-9)
Supplement: Supplementary file 1 — Supplementary Figures. [file 41598_2022_17473_MOESM1_ESM.docx]

**Supplementary Information**

**Title:** Coordination dynamics of thoracic and abdominal movements during voluntary breathing

**Author list:** Mimu Higashino, Kohei Miyata, and Kazutoshi Kudo

**Relative phase histogram for each participant and condition**


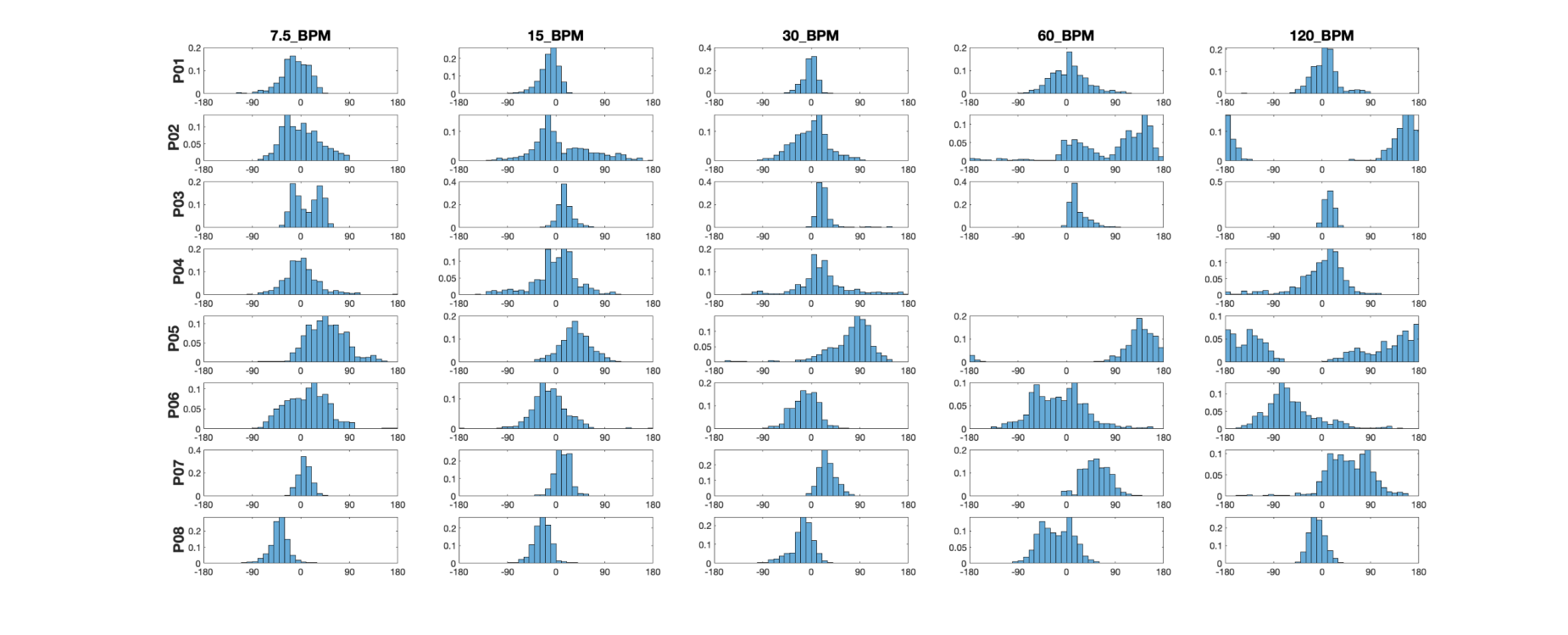

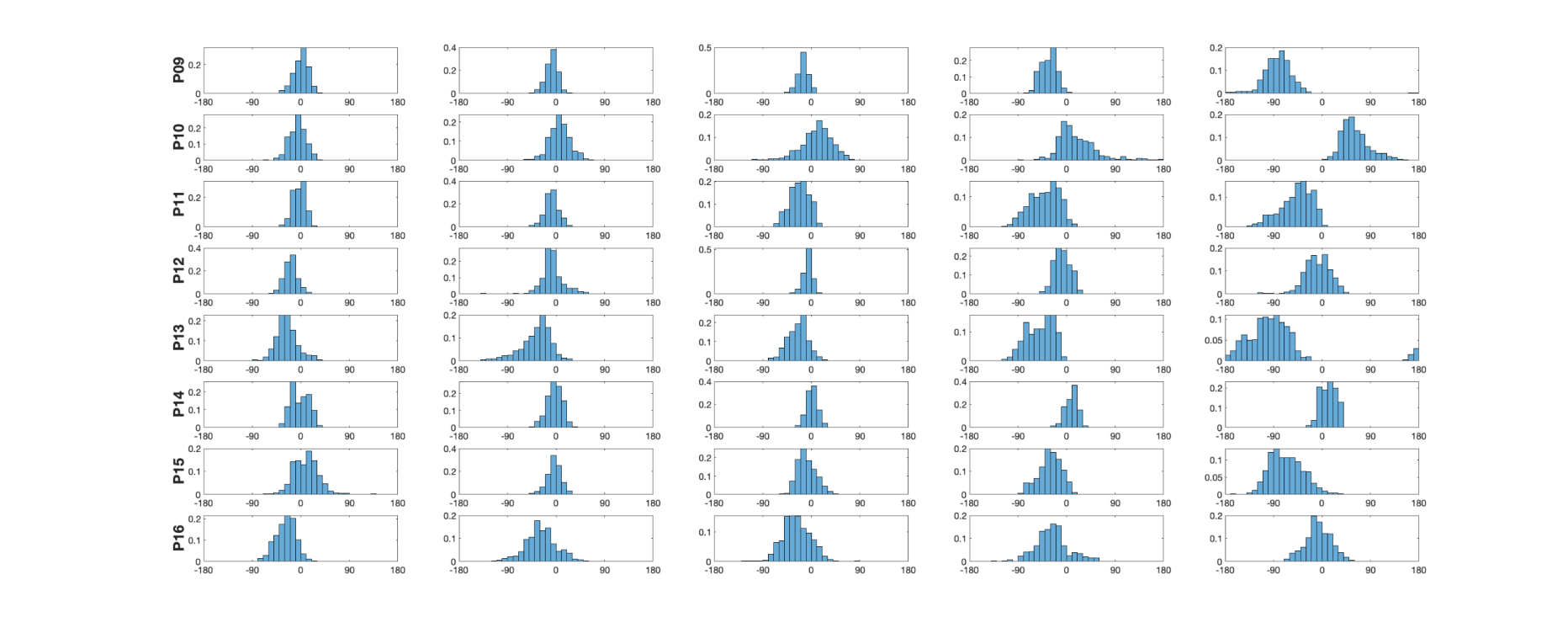


*Supplementary Figure S1.* The relative phase histograms for each participant and condition

Figure S1 shows relative phase histograms of all trials for each participant and condition. The y-axis indicates probability. Most of the relative phase showed unimodal distribution with single peak around the mean value, while a few bimodal distribution was observed. In our experiment, we didn't observe uniform distribution, which can be caused by phase wandering.

**The effects of breathing frequency on the amplitude of thoracic and abdominal movements**

Previous studies suggest that movement frequency affects the amplitude of rhythmic movements. To explore the effect of breathing frequency, we performed a 2-way ANOVA with two within-subject factors – breathing frequency and body parts (thoracic and abdominal) – on the ratio of amplitudes against the maximum amplitude across trials. We used the ratio because the unit of output from the strain gauge band of Hexoskin was arbitrary.


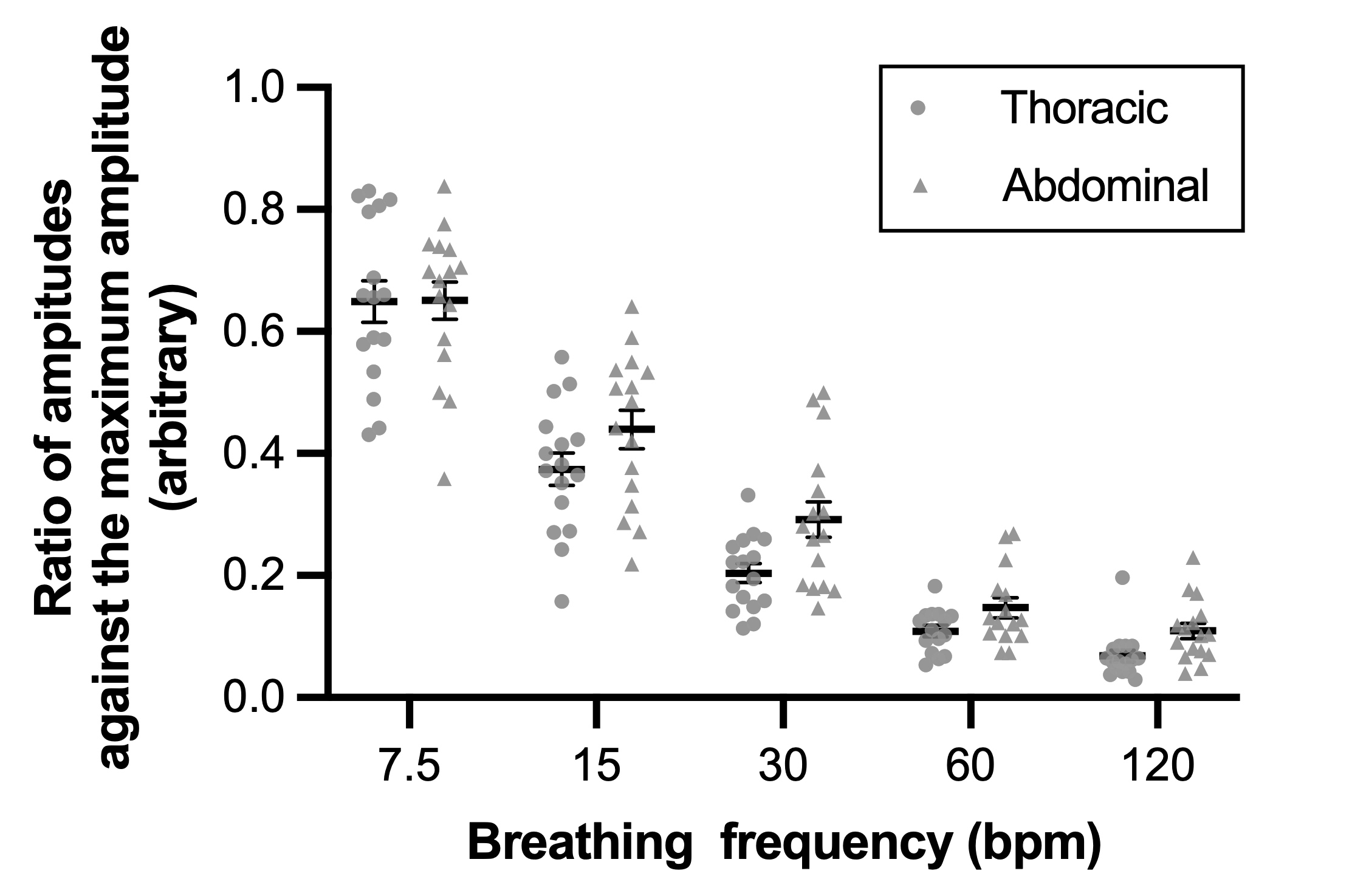


*Supplementary Figure S2.* The ratio of amplitudes against the maximum amplitude

The main effect of breathing frequency was significant, *F*(2.21, 30.92) = 266.45, *p* < .001, = .83. The main effect of body parts and interaction were not significant, *F*(1, 14) = 2.95, *p* = .108, and *F*(2.11, 29.55) = 1.85, *p* = .174, respectively. Therefore, our results indicated that breathing frequency decreases the amplitude of both thoracic and abdominal movements.
